# Methodology for Evaluating Process Mining Tools in IoT Contexts

**DOI:** 10.3390/s26031020

**Published:** 2026-02-04

**Authors:** Tilen Tratnjek, Gregor Polančič

**Affiliations:** Faculty of Electrical Engineering and Computer Science, University of Maribor, Koroška cesta 46, 2000 Maribor, Slovenia; gregor.polancic@um.si

**Keywords:** IoT, process mining, comparative analysis, task-based evaluation

## Abstract

As IoT environments continue to grow in scale and complexity, the increasing number of interconnected sensors and devices makes end-to-end system behaviour progressively harder to understand. Process mining offers strong potential to address this challenge by transforming sensor-driven event data into interpretable insights at the process level. Yet, current tools are typically designed for business processes, not sensor-driven IoT workflows, which raises questions about their suitability in the IoT context. This discrepancy is evident in existing comparative studies, which often rely on feature checklists, rarely consider usability and interaction effort, or fail to evaluate support for domain-specific analytical tasks. This study introduces a structured evaluation methodology that combines a functional capability assessment derived from vendor materials with a task-based evaluation grounded in 13 representative questions from an IoT-oriented smart factory scenario, focusing on clarity, ease of use, and the ability to address context-specific analytical needs. The results highlight notable strengths and trade-offs among the investigated tools, demonstrating substantial variation in usability, effort, and analytical coverage, and showing that no single tool fully supports the breadth of process-intelligence needs in IoT contexts. The proposed methodology provides a replicable foundation for evaluating process mining tools in domain-specific settings and supports more informed tool selection for IoT-driven analytical workflows.

## 1. Introduction

The integration of Internet of Things (IoT) technologies into industrial environments, particularly smart factories, has resulted in large volumes of event data being generated that can be used to analyse operational workflows. At the same time, as the number of interconnected devices grows, understanding how local events relate to end-to-end system behaviour becomes progressively more challenging. By applying process mining techniques, IoT engineers and analysts can gain a deeper understanding of process executions, identify bottlenecks, and check conformance to expected behaviour [1].

While IoT-enabled systems generate heterogeneous, high-frequency sensor data, transforming this data into discrete process events typically occurs upstream of process mining tools. The sensor aggregation and sensor-to-event abstractions are commonly handled by IoT data engineering and system integration layers (e.g., manufacturing execution systems), whereas process mining tools are designed primarily as analytical systems that assume the availability of structured event logs described in more detail in Section 2.1. Consequently, this study examines the analytical capabilities of current process mining tools when operating on process-level event logs derived from IoT-enabled systems, where the IoT context is primarily conveyed through the application scenario rather than direct sensor-level representations.

According to the systematic literature review by Kesici et al. [2], comparative studies of process mining tools tend to emphasise feature availability through binary checklists, while overlooking usability and the application of tools to realistic analytical tasks that reflect concrete domain questions. This study responds to these methodological gaps by evaluating three well-established process mining tools, Apromore Portal, Fluxicon Disco and ProM, from the perspective of IoT-oriented process analysis. Rather than addressing challenges related to raw sensor data or sensor-to-event abstraction [1], we focus on process-level event logs, which remain the primary format supported by current tools [3]. Using a preprocessed process-level event log derived from a physical smart factory, we assess how well the selected tools support common process mining tasks such as discovering process flows, analysing performance, and verifying execution against expected process behaviour in an IoT-specific analytical context scenario.

The comparative analysis followed a structured evaluation procedure that combined the analysis of vendor materials described in Section 3.3.3 with a task-based evaluation. Vendor materials were reviewed, and relevant claims were summarised and scored against predefined functional areas for each tool. For task-based evaluation, the process mining tools were applied to the same smart factory dataset and answered an identical set of analytical questions, thereby increasing the likelihood that the observed differences reflected the characteristics of the tools. The comparative analysis was guided by three research questions: RQ1 asks “how do the investigated tools differ in their functional capabilities, as identified from vendor materials and scored on defined criteria?”; RQ2 examines “how usable these tools are for IoT engineers when performing a fixed set of analytical tasks on smart factory event logs?”; and RQ3 investigates “how consistent the analytical outcomes are across tools on the same dataset?”. These questions provide the foundation for comparing the functionality, usability, and reliability of the results in IoT-driven contexts.

The novelty of this work lies in its evaluation methodology, which includes a structured framework that integrates qualitative assessment based on vendor materials and task-based evaluation that emphasises usability, clarity of outputs, and analytical coverage. This approach directly addresses the methodological gaps identified in the systematic literature review by Kesici et al. [2], which noted that previous comparative studies of process mining tools often lacked usability assessments and contextual applications of process mining tools.

The remainder of the article is organised as follows. Section 2 reviews previous work on process mining in IoT contexts and outlines the theoretical foundations that inform our study. Section 3 introduces the smart factory dataset, evaluated process mining tools, and details the evaluation framework. Section 4 presents the results of the comparative evaluation between the three tools. Section 5 interprets these findings, highlighting both practical implications and methodological limitations. Finally, Section 6 concludes the study and outlines the directions for future research.

## 2. Background and Related Work

Process mining connects with IoT by turning structured execution data derived from raw sensor inputs into process views. This shift from low-level monitoring to process-level analysis can allow engineers to diagnose, validate, and improve IoT-driven systems. The mining of smart environments processes helps detect inefficiencies, troubleshoot failures, and ensure that IoT infrastructures deliver practical value [1]. Building on these foundations, process intelligence extends the role of process mining by not only analysing past executions, but also enabling continuous monitoring and optimisation in real-time, helping systems remain both efficient and adaptable. It supports predictive and prescriptive analytics, allowing organisations to detect deviations early while anticipating and preventing potential problems [4]. By transforming raw IoT data into actionable insights, process intelligence, with process mining as one of its core components, closes the gap between data collection and informed decision making [5].

### 2.1. Process Mining Foundations

Process mining combines data, analytical techniques, and visualisation to provide an objective understanding of how processes are executed within an organisation. At its core lies the event log, a structured record of all process activities. Modern information systems such as Enterprise Resource Planning (ERP) platforms, which integrate and manage core business processes, and Manufacturing Execution Systems (MESs), which monitor and control production operations and automatically capture process steps, forming event logs [6,7].

**Figure 1:**
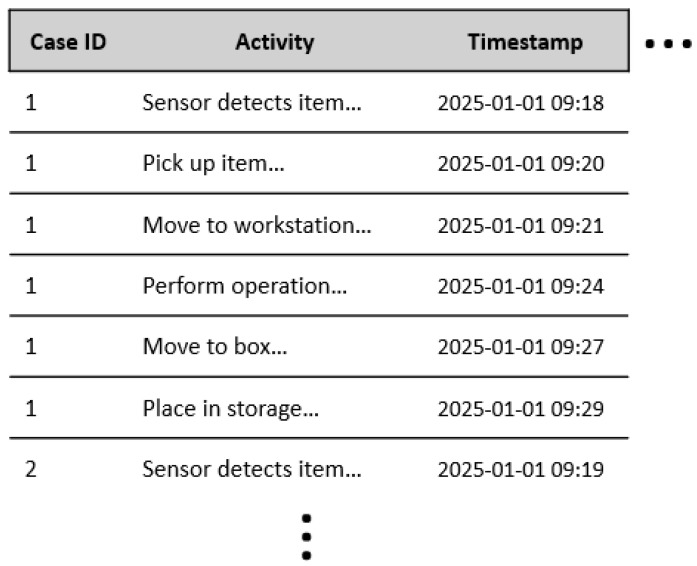
Example of process event log from a smart environment (illustrative, synthetic data). Real event logs may also include attributes such as resource, energy consumption, duration, etc. [1,6].

For a meaningful analysis, each event in a log must include three fundamental attributes. The case ID uniquely identifies the process instance, for example, a product batch number or workflow. The activity specifies the action performed, such as a robot arm assembling a component or a conveyor belt starting. The timestamp records the exact date and time of execution. Together, these attributes provide a structured foundation for understanding what happens in a process, when it occurs, and in what sequence, enabling data-driven insights instead of subjective assumptions [6,8]. An example of an event log from a smart environment is shown in Figure 1.

With the data collected, the process mining is commonly grouped into three core types: discovery, conformance checking, and enhancement. In discovery, event logs are analysed to automatically create a process model. Instead of engineers manually modelling workflows with traditional modelling tools, process mining tools show how the process actually runs based on the recorded event data. Process discovery typically yields process maps that consolidate individual events into a comprehensive view of the process, revealing primary execution paths, alternative flows, and unexpected loops. The process map is a key tool for understanding, as it transforms complex business logic into a clear visual form that is usable to both analysts and management. It serves as a foundation for further analysis and strategic decisions, allowing the identification of bottlenecks, inefficiencies, and opportunities for optimisation [6,8]. In conformance checking, the discovered process is compared with a reference model of how the process is supposed to run. This makes it possible to spot skipped steps, repeated actions, or rule violations. In an IoT setting, it also helps identify performance problems, such as machines that slow down the overall flow or activities that create long waiting times. For enhancement, insights from discovery and conformance can be used to improve the process. With clear evidence of where issues occur, engineers can take targeted action rather than guessing. In smart environments, this step is especially valuable because predictive analytics can warn about problems before they occur, allowing proactive maintenance and smoother production [6,7].

### 2.2. Process Mining in Smart Environments

The integration of IoT with process mining opens up new dimensions of insight into business process management. Smart environments are characterised by a wide range of connected devices that enable continuous monitoring. IoT devices capture machine statuses, physical movements, and environmental conditions, providing real-time insights into system performance. The integration of sensors into workflows enables activities that were previously opaque, particularly manual or physical tasks, to become visible at the process level. This creates opportunities for proactive monitoring, real-time compliance checks, and flexible system behaviour that better reflects actual implementation [1].

Process mining has often been used in organisational settings where activities are recorded by information systems. These contexts provided structured event logs, but often offered only partial insight, as manual or physical activities were under-represented. The IoT expands the scope of application by enabling more direct recording of events in sensor-rich environments. Sensors, machines, and connected devices generate high-frequency data that can be converted into event logs, making them suitable for process mining. Compared to traditional logs, events obtained from the IoT can capture more detailed and accurate information while reducing the reliance on manual entry. However, the volume and diversity of IoT data pose challenges, including quantity, level of detail, and noise, which must be addressed through preprocessing and integration [1,9]. Although traditional IoT monitoring focuses on low-level signals, such as sensor readings, machine states, or event triggers, it often lacks visibility into how complete processes unfold over time. This is especially problematic in complex factory environments, where machines interact across workflows and failures or inefficiencies only become visible when viewed at the level of full process execution. This gap motivates the use of process mining not as a business analytics tool, but as a method for diagnosis and validation in IoT-based systems [1].

In this work, we take the perspective of an IoT engineer who works with structured process execution data, derived from raw sensor inputs through a workflow management system. We do not analyse raw sensor data as they were beyond the scope of this study due to the limited support that current process mining tools provide for continuous IoT data [3]. Instead, we evaluated how well current process mining tools support the analysis of logs that reflect IoT-driven processes. Our focus is on clarity of results and usability. The development of IoT applications is a multidisciplinary process in which technical knowledge alone is insufficient [10]. An IoT engineer must also be process-aware, understanding how the system contributes to the broader workflow and supports the operational or business objectives. As Reinkemeyer [11] emphasises, technological solutions only create value when aligned with organisational needs and result in a measurable impact; otherwise, even advanced systems risk failing to deliver value in their intended context.

Our focus was on evaluating the capabilities of existing tools rather than implementing new IoT data pipelines. This approach enabled us to make a realistic assessment of how current process mining tools perform on IoT-derived event data. The observed limitations, therefore, highlight current technological boundaries and guide future development of IoT process intelligence solutions.

### 2.3. Process Mining Tools

To perform all the steps of the process mining described in Section 2.1, specialised software tools are needed. These tools process event logs, apply process discovery algorithms, verify conformance to defined models, and support process analysis and improvement. In addition to core functions, they may also offer advanced features, such as integration with external systems or sophisticated methods like AI-based prediction or classification.

Process mining tools differ in their focus and maturity [12]. Commercial tools are typically designed for business adoption, whereas open-source and academic tools, such as ProM, are often used in research settings. Based on an analysis of ProM 6, it can be seen that it includes a large number of process mining algorithms and provides an architecture that facilitates the development of new plug-ins and extensions [6,13].

Despite their maturity in organisational contexts, current process mining tools face notable limitations when applied to IoT environments. IoT data is heterogeneous, high-volume, and often weakly structured, making it difficult to transform it into event logs suitable for process mining. Unlike enterprise data, sensor readings and machine signals lack standardisation, and no unified approach exists for semantically enriching them with contextual information. As a result, many implementations rely on proprietary formats, which harms interoperability [3,10]. SensorStream, a XES extension [3] was proposed as a way to standardise and enrich sensor data within event logs. However, it has not yet reached widespread tool support, which limits its practical use in IoT-focused analyses. Moreover, logs enriched with detailed sensor data can grow rapidly in size, raising concerns about scalability that existing tools may not yet be equipped to handle [3].

### 2.4. Related Work

Before conducting the comparative analysis, we examined how previous studies had structured their comparisons of process mining tools. This provided insight into the methodological approaches employed, the comparison criteria used, and how the functionalities of the individual tools were evaluated. Additionally, reviewing the literature helped us identify frequently compared tools and spot potential gaps in existing studies. This information was crucial in justifying our selection of tools and the design of the evaluation framework. This search ensured the methodological reliability and relevance of the analysis.

We conducted a systematic search for related literature using the Google Scholar database, applying the query intitle:(“process mining”) AND intitle:(comparison OR “comparative analysis”) and limiting the results to the period 2019–2025 to capture the most recent developments, as process mining tools evolve and older evaluations may no longer reflect current capabilities. In the first phase, we filtered the results based on recency, type of research, and relevance to the content, including only articles that compare the functionality of the process mining tools. We excluded papers that focused solely on theoretical aspects without addressing specific process mining tools. Based on this, we discarded most of the results and identified 18 studies as potentially relevant. In the second phase, we reviewed these in more detail and further evaluated them based on a direct comparison of tools, relevance, and the availability of full-text resources through the University of Maribor.

From the search, we identified the systematic review of the literature [2] that provided an important foundation but also revealed key gaps. It showed that most evaluations emphasised technical functionality while overlooking usability and user experience, that binary evaluations dominated, and that contextual factors such as specific perspectives were rarely considered. Building on this foundation, our study seeks to partially close these gaps. We extend previous work by introducing a more qualitative and task-based evaluation approach that not only considers functional coverage but also examines dimensions such as effort, ease of use, and clarity.

In addition to reviewing comparative studies, we also investigated research addressing the integration of process mining and the IoT. We did not identify relevant comparative evaluations in our search. However, we found several conceptual contributions that frame the challenges of bringing process mining to IoT environments, such as [1,10]. These contributions do not directly assess the tools, but frame the broader context, showing why IoT remains largely absent in current evaluations.

In the reviewed literature, there is no standardised methodology or a uniform set of criteria to compare process mining software tools [2]. Most studies share a focus on core functionalities such as process discovery, conformance checking, and performance analysis, while evaluations that consider contextual factors or ease of use of tools remain scarce [2,14,15]. The identified gap that we address in this work with the proposed methodology. Data management capabilities, including event log import and transformation, are also common evaluation factors. However, the reviewed studies diverge in scope and methodology [15]. The criteria used range from binary and qualitative descriptions [16,17] to weighted approaches or decision trees [14]. The number and type of tools compared also differ, ranging from focused case studies on a few applications to reviews of multiple platforms. Although most of the evaluations target general business use, there is a notable lack of frameworks tailored to specific domains [2]. This study does not evaluate tools from a business decision perspective. Instead, it focuses on methodological and analytical suitability, examining how process mining tools support specific analytical tasks, usability aspects, and functional capabilities when applied to IoT-derived process event logs.

## 3. Materials and Methods

The review of existing studies showed that most comparisons of process mining tools emphasise technical functionality, while aspects such as usability, user effort, or contextual application are less frequently addressed [2]. We also found that comparative evaluations rarely consider IoT settings, although the existing manifesto [1] has highlighted the potential to apply process mining in such environments. This section describes the IoT system and the dataset used, the selected process mining tools, and the evaluation framework applied in the comparative analysis of the investigated process mining tools. We then outline the functional areas assessed, the task-based evaluation criteria, and the benchmarking protocol. Finally, we specify the testing environment and the artefacts provided for transparency and reuse.

### 3.1. IoT System and Dataset

**Figure 2:**
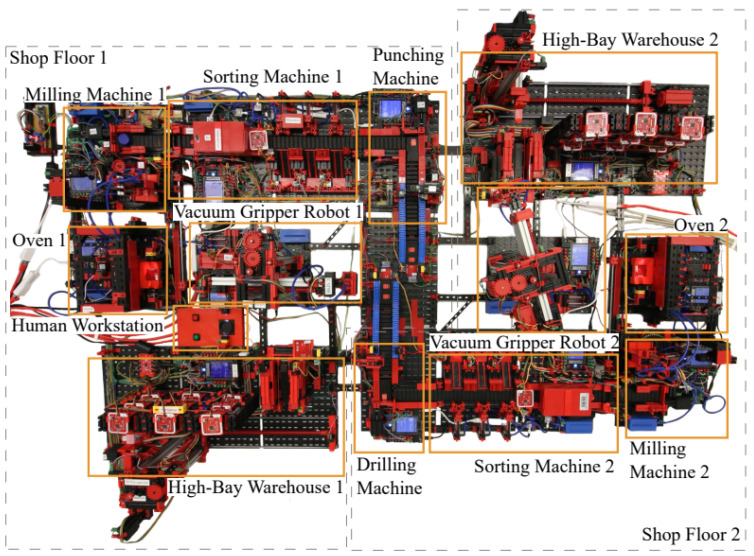
The Physical Factory Simulation Model [19].

The event logs used originate from a smart factory model based on Fischertechnik hardware, as described in [18]. The factory consists of two production lines, each with six identical machines. In addition, each line includes distinct machines: a punching machine and a human workstation in the first, and a drilling machine in the second [18]. The setup is presented in Figure 2. Details regarding raw data generation, sensor configurations, communication protocols, and the abstraction from sensor signals to event-level activities are described in [18].

The system executes 16 different processes over a runtime of more than 20 h, with process execution coordinated through a Workflow Management System that follows a service-based architecture. During runtime, data is captured from three primary sources. The first source consists of sensor and actuator data, which is streamed via middleware into Apache Kafka. The second source is the set of execution traces generated by the Workflow Management System itself. The third source is the collection of web server logs, which provide enriched contextual information such as planned versus actual execution times and the causes of exceptions [18]. Because the evaluated tools do not provide support for the SensorStream XES extension and thus cannot process sensor metadata [18], we restricted our evaluation to process-level event data with standard attributes (activity, timestamp, case, resource). For evaluation, we used the cleaned (corrected) version of the event log. This version applies all preprocessing steps (e.g., deduplication, missing value imputation, timestamp correction) [18] and reflects the structured log format that an IoT engineer would typically produce before analysis. Although high-quality event data from operational IoT systems remains scarce, the dataset used in this study reflects the type of structured event logs commonly prepared for analysis in IoT settings. Simulated or synthetic data was not used, as doing so could introduce artificial patterns or biases that may unintentionally influence tool performance results. Our aim was to assess how the current state of process mining tools supports IoT engineers under realistic conditions, based on the data characteristics that they would encounter in practice.

### 3.2. Evaluated Process Mining Tools

The market offers a wide range of process mining tools [20] that differ in functionality, accessibility, and technical architecture. To ensure a fair and representative comparison, we applied clear selection criteria. Specifically, we limited our analysis to three tools that cover the enterprise-grade, commercial, and open-source categories of the market, and that provide academic or community licenses to allow for consistent evaluation under comparable conditions. Tools that rely on proprietary query languages were excluded to maintain fairness. Based on these criteria, we selected Fluxicon Disco, Apromore Portal, and ProM, as they are well-established, widely referenced in prior comparative studies, and cover complementary market segments of the process mining ecosystem.

The selection of these tools is not intended to represent an exhaustive comparison of the entire market, nor to generalise results to entire classes of process mining software. Instead, the goal is to examine illustrative instances of tools with different design orientations and development backgrounds: an open-source, research-oriented framework (ProM), a commercial desktop tool (Fluxicon Disco), and an enterprise-grade, cloud-based platform (Apromore). Differences in release cycles, update frequency, and UX maturity are therefore acknowledged as characteristics of the selected tools rather than of their broader categories.

#### 3.2.1. Apromore Portal

Apromore Portal 10.3 (hereafter Apromore) is a commercially licensed process mining tool developed by Apromore Pty Ltd (4C/700 Swanston Street, Carlton, Melbourne, VIC, Australia). It originated from a research collaboration between the University of Melbourne and the University of Tartu. Since its initial release in 2009, the tool has undergone continuous development, with the latest update arriving in March 2025. Users can access the service through web browsers as cloud-based software or by installing it on their own infrastructure [21]. In addition, Apromore has been recognised in Gartner’s 2025 Magic Quadrant [12] as one of the leading enterprise process mining platforms, ensuring that enterprise-level capabilities are represented.

**Figure 3:**
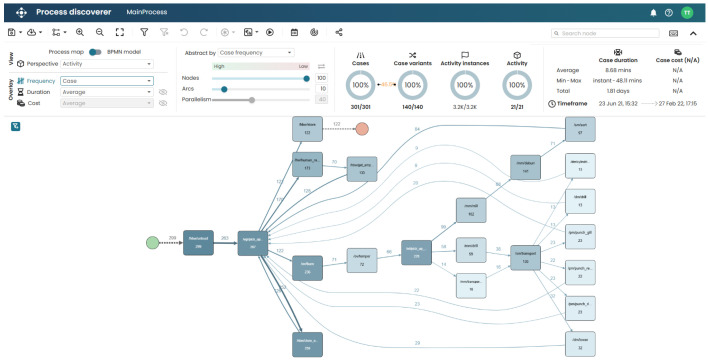
Illustrative example of a discovered process model in Apromore Portal version 10.3, shown to provide an impression of the user interface [21].

The tool is designed for business process analysis and optimisation, offering features such as process discovery, conformance checking, simulation, and predictive analytics. Part of the user interface showing a discovered process map is shown in Figure 3. Apromore is based on over a decade of innovation and academic research, including the results of several PhD theses and more than 200 scientific publications. Although the tool initially started as an open-source project, it now operates as a commercial product with flexible pricing models based on usage scale, and it offers technical support and regular updates [21].

#### 3.2.2. Fluxicon Disco

Fluxicon Disco 4.1.9 (hereafter referred to as Disco) is a commercially licensed process mining tool developed by Fluxicon BV (Torenallee 20, 5617 BC Eindhoven, The Netherlands), a company founded by former academics with extensive experience in process mining. Since its initial release in 2011, Disco has undergone continuous refinement, with the most recent update in January 2025. The software runs on Windows (version 8 or later) and macOS (10.15 or later) and is distributed as a desktop application [22].

**Figure 4:**
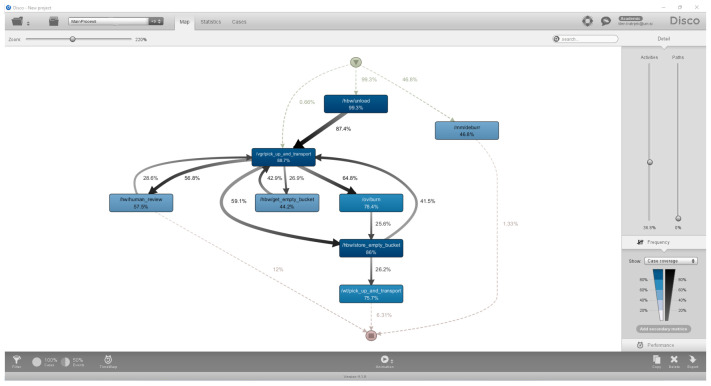
Illustrative example of a discovered process model in Fluxicon Disco version 4.1.9, shown to provide an impression of the user interface [22].

Disco is designed for professional process analysts, offering automated process discovery, log filtering, performance analysis, and visualisation features. Its interface emphasises speed and usability, allowing users to import raw event logs and generate process maps in minutes. The tool includes high-performance filtering capabilities, animated process map replay, and detailed statistical views, making it suitable for exploring case variants, activity frequencies, and performance metrics. Disco supports standard event log formats and provides export functions for integration with other tools [22]. Figure 4 shows part of the user interface with a discovery process map.

#### 3.2.3. ProM

ProM 6.14 is an open source process mining tool developed by the Eindhoven University of Technology (TU/e). It was first released in 2004 as ProM 1.1, with the latest update in September 2023. The software runs on Windows, Linux, and macOS operating systems [23,24].

**Figure 5:**
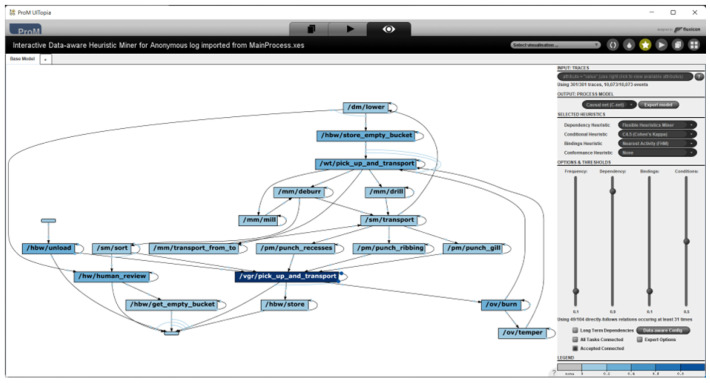
Illustrative example of a discovered process model in ProM 6.14 with Heuristic Miner, shown to provide an impression of the user interface [24].

The tool is intended for research and process analysis, focusing on process discovery, conformance checking, and process improvement based on event logs. ProM is known for its modular design, which allows the customisation of methods and the addition of new algorithms. It supports various process mining techniques, including Alpha Miner, Heuristic Miner, Fuzzy Miner, and Genetic Miner. The tool is built in Java and operates as a local application [13,23]. A part of the user interface showing a discovered process map is shown in Figure 5.

### 3.3. Evaluation Framework

Our evaluation framework was informed by two established theories from the field of information systems research. The Technology Acceptance Model (TAM) [25] highlights perceived usefulness and ease of use as key factors in technology adoption. Although our study did not measure user perceptions, these concepts informed our approach. Ease of use influenced our scoring of cognitive effort and interface clarity, as well as whether the tools enable IoT engineers to complete analytical tasks effectively. The Task-Technology Fit (TTF) theory [26] offers a complementary perspective, emphasising that technology generates value only when its functionality aligns with the tasks of its users. This idea underpins our task-based evaluation and the functional areas against which the tools were evaluated. Although we do not formally test TTF, the framework helped us structure the evaluation around concrete tasks rather than binary feature lists.

This evaluation framework assesses how well process mining tools support the analysis of IoT-driven process data. Most comparative analyses emphasise feature lists, but neglect user roles and task requirements [2], which restricts their relevance in applied contexts. We designed a structured framework to address this. It compares tools not only by features, but by how well they support real tasks from an IoT engineer’s point of view, demonstrating both what vendors claim their tools can do and how effectively they achieve it.

#### 3.3.1. Functional Capabilities

Functional capabilities describe the range of features that process mining tools provide to support core activities such as data management, process discovery, conformance checking, performance analysis, and reporting [6]. We defined six functional areas based on established literature, particularly the work of van der Aalst in Process Mining: Data Science in Action [6] and the Process Mining Manifesto [27]. In these sources, process mining is conceptualised as a sequence of stages that connect event data to process insights. Building on this perspective, we derived six functional areas that reflect the essential capabilities a process mining tool should provide: (1) data management, (2) process discovery, (3) conformance checking, (4) process analysis, (5) monitoring and reporting, and (6) support. The first five areas correspond directly to the core phases and functionalities described in the foundational literature [6,27] and related works mentioned in Section 2.4. The sixth area of this study accounts for aspects that influence the adoption of tools, such as documentation quality, community support, and user guides; these can be related to the enablers identified by Reinkemeyer [11], who emphasises training, community building, and knowledge sharing as key drivers of user adoption. Table 1 provides descriptions of the six functional areas defined in this study. These areas form the analytical foundation for the qualitative assessment, based on vendor materials, and define the dimensions through which the process mining tools were evaluated.

Unlike the raw functionality counts used in related work, as observed by [2], which treat all functions as binary (present or absent), our evaluation takes a structured qualitative approach, focusing on the extent to which functional areas are implemented. A tool that implements more features may appear stronger than one that offers fewer but more robust capabilities, which is misleading. Moreover, the raw-count approach assumes uniform importance across all features, although the practical value of each function varies by user and use case, as also observed in [2].

To ensure a transparent and reproducible scoring method, relevant information was collected for each functional area defined in Table 1 from official vendor sources, including websites, documentation, user guides, and tool interfaces. The summarised descriptions of the collected information were then assessed against the evaluation criteria, and each functional area was assigned a rating from 0 to 5 based on the evaluator’s expert interpretation of how well the documented capabilities corresponded to the levels defined in the scale. After assigning a score, a justification was written to clarify why the observed functionality corresponded to the assigned score. An illustration of this scoring approach is provided in Section 4.1, and the complete scoring templates with all the summaries, references, and justifications are available in the public repository [28].

Because our assessment reflects the perspective of IoT engineers, a fully objective weighting scheme would not remove this inherent domain bias. Using a qualitative approach with transparent justifications, our methodology remains adaptable, allowing evaluators from other roles or contexts to reinterpret the assessments of functional capabilities according to their own priorities and perspectives. The selection of a process mining tool is inherently context-dependent: factors such as ease of use, required expertise, and the user’s role strongly influence what “best” means in practice.

#### 3.3.2. Task-Based Evaluation

This part of the evaluation assesses how effectively process mining tools support analysts in practice. Although the analysis of vendor materials identifies the features claimed by the vendor, it does not reveal how effectively they can be applied in real analytical work. To address this, we evaluated the tools using three complementary dimensions: effort, ease of use, and clarity. These dimensions capture both the workload imposed by the tool and the clarity of its outputs, which is particularly important in IoT-driven contexts where engineers may not be experts in process mining.

Effort was measured by counting the number of user interactions, primarily mouse clicks, required to complete each analytical task. While this metric does not capture the full depth of user effort, it provides a simple and objective proxy for the interaction workload imposed by a tool. Effort was operationalised as the minimal click path required to obtain a result. The evaluator first explored available options to identify a viable solution and then re-executed the optimal path once to confirm the minimal click count. Only interactions that triggered a user-interface action were included, and keyboard shortcuts were excluded to ensure consistency across tools. Minimal click counts were intentionally chosen because they are less sensitive to evaluator familiarity, learning effects, and experimental conditions than time-based measures. In addition, effort scores can be combined with qualitative assessments, such as ease of use and clarity, to explore correlations, for example, whether tools that require fewer clicks also deliver clearer output or feel easier to operate.

The ease of use reflects how intuitive and usable a tool is to complete analytical tasks [25,29]. Many comparative studies focus heavily on feature lists while neglecting usability [2], yet ease of use often determines whether a tool can be adopted successfully. We therefore defined a structured five-point scoring framework (Table 2), ranging from 1 (very hard to use, requiring high cognitive effort and technical knowledge) to 5 (very easy, with intuitive, consistent, and self-explanatory workflows). Scores were assigned based on interface clarity, labelling, workflow consistency, and the overall cognitive effort required to complete tasks.

Clarity measures how easily an analyst can interpret and extract insights from outputs such as visualisations or tables. Even accurate data loses value if presented in a confusing or poorly structured way. Clear presentation reduces cognitive load, minimises misinterpretation, and supports efficient decision making [30]. The results were scored on a five-point scale (Table 3), ranging from 1 (difficult to interpret, poorly labelled, and confusing) to 5 (immediately understandable, with strong structure and clear labelling).

A task was considered successfully completed if the evaluator could obtain an unambiguous answer using the tool within a reasonable amount of effort. This includes cases where the tool did not directly output the final result but provided sufficient intermediate information from which the answer could be derived through simple manual actions by the analyst (e.g., counting elements in a visualisation). Tasks for which the tool did not provide adequate support for deriving the answer, even with reasonable manual effort, were classified as unfeasible and are marked with “/” in the tables. This includes cases where answering the question would require unsupported transformations or external preprocessing that has not been evaluated in this study.

In addition to usability, the task-based evaluation also enabled derivative analysis, which investigates how consistent the analytical outcomes are between the investigated tools on the same dataset. Consistency was assessed by comparing whether different tools produced broadly similar answers to the same analytical questions without requiring exact alignment, as small differences due to rounding were tolerated. With task-based evaluation, it was also recorded which tools were capable of answering which questions, since gaps in coverage can be as important as differences in reported values. This approach is preferable because it reflects the realities of practical tool use, where analysts are primarily concerned with whether tools yield comparable insights and provide coverage of the required analyses, rather than with exact numerical agreement.

#### 3.3.3. Benchmarking Protocol

The evaluation followed a two-stage protocol that combined the analysis of vendor materials with task-based evaluation. This design ensured that both the vendor’s claimed functionality and practical usability were systematically assessed. Vendor sources, including manuals, feature lists, technical specifications, and official websites, were analysed to establish the functional capabilities claimed by each vendor. For each functional area, descriptions were summarised and translated into standardised scores using the criteria in Table 4. The scores ranged from 0 (no support) to 5 (advanced functionality). In addition to scores, justifications were recorded to maintain transparency and replicability. Where features did not fit predefined levels, limited flexibility was applied to avoid penalising design differences while ensuring consistency.

To complement the review, each tool was applied to the same smart factory dataset and answered an identical set of 13 analytical questions. The dataset and the practitioner were kept constant so that the observed differences could be attributed more likely to the tools themselves. To minimise evaluator bias, the analysis was conducted by an expert evaluator, and the results were subsequently audited by a senior expert evaluator. Performance was assessed along the three dimensions of usability introduced in Section 3.3.2: effort (number of interactions required), ease of use (cognitive workload and interface guidance), and clarity (interpretability of outputs). Observed behaviours were recorded in structured templates available in the public repository [28], which captured the number of steps, qualitative notes, screenshots, and assigned scores. The analytical questions (Table 5) covered process discovery, conformance, performance, and resource perspectives relevant to the IoT-driven smart manufacturing factory.

The senior expert conducted an audit of the evaluation materials, focusing on the clarity of score interpretation, justification, and alignment with the evaluation criteria. Where scores or justifications appeared atypical or insufficiently supported, individual tasks were selectively reviewed in more detail. The purpose of this audit was to enhance the consistency and transparency of qualitative judgments. Feedback primarily resulted in refinements of textual justifications, and in a small number of cases, minor score adjustments were made, limited to a maximum of one point on the 0–5 scale. Because the review did not involve a parallel reassessment of all items and adjustments were made ad hoc, quantitative inter-rater agreement measures are not applicable, and no claims are made regarding agreement frequencies or disagreement patterns.

The selection of the 13 analytical questions is grounded in established process mining literature and reflects the core phases of the process mining life cycle, encompassing discovery, conformance, performance, and resource analysis. Consistent with the Process Mining Manifesto [27], which emphasises that process mining activities should be driven by analytical questions, the selected questions define the analytical focus of the evaluation rather than the data extraction process. Rather than being exhaustive, the questions were designed to represent essential analytical tasks that a practitioner would expect a process mining tool to support in an IoT-driven manufacturing context.

The typical analytical questions were designed around the smart factory dataset to cover the main perspectives of process mining relevant to IoT-driven manufacturing. Q1, Q2, Q4, Q8 and Q10 focus on process discovery, while Q3, Q5, Q6, Q7, Q11 and Q12 address performance analysis. Q9 examines conformance checking, and Q13 targets the resource perspective. The questions vary in difficulty, with simpler tasks, such as counting variants Q2, contrasted with more demanding analyses, such as conformance checking Q9. Discovery and performance are represented more heavily because they align more closely with the structure and richness of the dataset, while conformance and resource-related tasks are covered by fewer but carefully selected questions. This balance ensures that the evaluation captures both the variety of process mining perspectives and the practical realities of applying them to IoT event data.

### 3.4. Evaluation Environment

All task-based evaluations were performed on a workstation equipped with Windows 11 Pro 64-bit, an AMD Ryzen 7 4700U processor, and 16GB of RAM. Disco 4.1.9 and ProM 6.14 were installed in their latest available versions. Apromore 10.3 was accessed via browser-based cloud platforms under an academic trial license, while ProM and Disco were run locally, the latter being run under an academic license.

Both phases of the evaluation were supported by structured templates to ensure consistency and replicability. In the analysis of vendor materials, a template was used to record summaries of the stated functional capabilities of each process mining tool, the assigned scores, and the justifications for these scores. During task-based evaluation, a second template was used to record the steps of the task, the number of interactions, the potential problems encountered, and qualitative observations about clarity and usability. The recorded templates, which contain all the scores and evaluator notes, are published in an online repository [28]. Minor editorial polishing was applied to improve readability, but the content remains an authentic record of the evaluation process.

Additionally, GenAI was used during the writing of this work to enhance sentence clarity, readability, and language refinement. After applying GenAI, the authors verified and retained full control over the accuracy and coherence of the content.

## 4. Results

Based on the research conducted, we obtained the following results, which are presented below in a structured manner according to the research questions posed to ensure clarity and alignment between the study design and the findings. Accordingly, the reported scores reflect tool-specific observations within the defined evaluation context and should not be interpreted as overall rankings.

### 4.1. Functional Capabilities Based on Analysis of Vendor Materials

This subsection addresses RQ1, which inquires about the differences in functional capabilities identified among the investigated tools based on vendor materials. The results of the functional capabilities based on the review of the documentation are presented in Table 6. The methodology for the analysis of vendor materials is described in Section 3.3.3, while limitations are discussed in Section 5.4.

Table 6 shows that Apromore scored the highest in all functional areas, particularly in monitoring and reporting, where Disco and ProM scored lower. ProM demonstrated strong capabilities in process discovery and conformance checking, but scored lower in areas such as support and monitoring. Disco excelled in process discovery and support, but showed weaker performance in conformance checking and monitoring. These differences highlight the varying strengths and weaknesses of the tools as documented by their vendors.

To illustrate our evaluation procedure in detail, we focus on the capability of monitoring and reporting. This dimension was selected because it is particularly relevant in IoT contexts and because the investigated tools demonstrated the greatest divergence in this area. The review examined the extent to which tools support automated reporting and live monitoring. Apromore received full support due to its dashboards, key performance indicator (KPI) tracking, automated reporting, and predictive monitoring features, which align well with the needs of IoT-driven environments where streaming data and continuous monitoring are critical. Disco, on the contrary, offered only basic support. Although it allows for the manual reimport of updated logs and provides snapshot reporting through export options, it lacks automation and real-time dashboards, restricting it to batch-oriented analysis. ProM scored lowest, with only static reporting through plug-in visualisations and exports and no functionality for continuous monitoring or automated reporting. This disparity between the three tools illustrates how our criteria were applied. The documented summary of functional capability was mapped against the scoring framework, scores were assigned and justifications were captured in structured templates that are publicly available in an online repository [28]. The same procedure was applied to all remaining capabilities, which are presented in summary form in Table 6.

### 4.2. Results of Task-Based Evaluation

This subsection addresses RQ2, which examines how usable the tools are for IoT engineers when performing a fixed set of analytical tasks. In the task-based evaluation, the evaluator used each investigated tool to answer the 13 analytical questions defined earlier, based on the smart factory event log. Each tool was evaluated in three complementary dimensions: clarity of outputs, effort (measured as the number of clicks), and ease of use. Together, these dimensions show not only whether tools can answer the questions, but also how interpretable the answers are to an IoT engineer.

Table 7 presents the clarity scores for all tasks. The results show that, overall, Apromore and Disco produced outputs that were easier to interpret, while some of ProM’s outputs were comparatively less clear.

The interaction effort required for each task is summarised in Table 8. In contrast to clarity, which measures how understandable outputs are once generated, effort reflects the workload imposed on the analyst through the number of interactions required. Here, clear differences between the tools emerged. Apromore consistently required the fewest clicks per question, Disco generally fell in the middle, and ProM almost always demanded the highest number of interactions. For example, in Q7 (cases longer than twice the average case duration), Apromore required only six clicks, while Disco required fifteen and ProM twenty-six. These figures demonstrate that the workload of interaction can vary substantially. Other results can be viewed in more detail in our publicly available evaluation materials [28].

The ease of use was assessed as the overall usability of the workflows, considering navigation, consistency of interactions, and how intuitively results could be obtained. Table 9 summarises the scores. Apromore and Disco generally achieved higher scores, reflecting polished interfaces and guided workflows. ProM displayed lower scores, as tasks required navigating across fragmented menus and filters.

Apromore required, on average, 5.5 clicks per task, compared to 12.7 for Disco and 18.9 for ProM. Ease of use scores followed the same trend: Apromore achieved the highest mean score of 4.9, Disco averaged 4.6, and ProM scored the lowest at 2.7. Clarity showed less variation across the tools. Apromore scored highest, averaging 4.9, followed by Disco at 4.8 and ProM at 4.1. In terms of coverage, Apromore could not answer two questions (Q5 and Q11), Disco could not answer four (Q4, Q5, Q9, and Q11), and ProM could not answer four as well (Q3, Q9, Q11, and Q12).

## 5. Discussion

This discussion interprets the findings of both the vendor materials analysis and the task-based evaluation, as well as the findings of related work. Together, they highlight not only the functional breadth claimed by vendors but also how these capabilities play out in practice when tools are applied to relevant analysis tasks. The following subsections first address insights from the analysis of vendor materials and then turn to the results of the task-based evaluation.

### 5.1. Claimed Functional Capabilities Based on Analysis of Vendor Materials

RQ1 examined the differences in functional capabilities of the investigated tools as identified in vendor materials. In particular, during the analysis of vendor materials, no explicit references to IoT were identified. The absence indicates that, at least from the perspective of reviewed materials, IoT is not yet considered a core functional dimension of these tools. This indicates that current tools still depend on preprocessed event logs and leave the burden of mapping raw sensor data to process-level events with the user. As highlighted by Janiesch et al. [1], one of the hardest problems in combining IoT with process analysis is to bridge the abstraction gap between raw sensor data and higher-level process events. As also noted in work [3], this abstraction gap remains a central challenge in IoT process analysis.

The divergence in monitoring and reporting capabilities underscores why current tools often fall short in IoT contexts, where continuous insights are crucial. Apromore was the only tool to support dashboards, KPI tracking, automated reporting, and predictive monitoring, features that align more closely with the needs of sensor-rich environments. However, even these capabilities remain tied to preprocessed logs and a lack of real-time integration, limiting their applicability in IoT-driven settings. Disco and ProM, by comparison, relied on manual log imports and static exports. From a smart factory perspective, where automated data capture makes continuous monitoring a baseline expectation rather than an advanced feature [1], these limitations indicate that none of the evaluated tools can yet provide full IoT process intelligence.

As an illustrative example of how functional capability scores were assigned, Disco received a low score for conformance checking because it does not support direct model-to-log comparison. According to the inspected documentation, Disco “does not provide formal model-versus-log conformance checking” [28] and instead relies on indirect exploration through filtering of discovered variants. As a result, structured techniques such as token replay or alignments are unavailable, which justifies the lower score.

### 5.2. Task-Based Evaluation

RQ2 examined how usable the tools are for IoT engineers when performing a fixed set of analytical tasks. The documentation review provided an overview of the functionality claimed by the vendors, while the task-based evaluation showed how the tools performed in practice on smart factory event logs. Differences in clarity, effort, and ease of use revealed distinct patterns in how the investigated tools support task execution. The discussion of the results for RQ2 is presented in Section 5.2.1.

Task-based evaluation also provided the basis for addressing RQ3, which investigated the consistency of analytical outcomes between tools on the same dataset. Consistency was assessed by comparing whether the tools produced broadly similar outputs and by noting which analytical questions each tool could answer. The discussion of the results for RQ3 is presented in Section 5.2.2.

#### 5.2.1. Usability of the Investigated Tools

The ease of use results showed a clear divide between Apromore and Disco on the one hand and ProM on the other. Apromore consistently offered better workflows and intuitive navigation, leading to the highest average score of 4.9. Disco was slightly less consistent, with some tasks requiring additional filtering or less obvious menu paths, but still maintained a strong overall score of 4.6. In contrast, ProM relied heavily on fragmented menus and plug-in configurations, which made even simple tasks more cumbersome, resulting in an average score of 2.7. These results suggest that while Apromore and Disco are usable to engineers without deep process mining expertise, ProM demands considerably more cognitive effort. In IoT-driven environments, where IoT engineers may not be process specialists, this usability gap represents a practical barrier to adoption.

The clarity evaluation revealed relatively consistent strengths for Apromore and Disco, both of which produced outputs that were easy to interpret across most tasks. The scores were close to maximum on discovery-oriented questions such as Q1, Q2, Q8 and Q10, where both tools provided well-labelled and visually structured results. ProM produced valid outputs, but often in formats that were less user-friendly. For example, in Q8 (longest path), Apromore and Disco displayed activity counts directly, but ProM required manual counting of activities from the visualisation, although the result was not produced directly. The task was still considered successfully completed, as the required effort remained reasonable and the answer could be derived unambiguously. These differences reduced ProM’s scores, particularly on tasks where complex tables or detailed process paths were involved. Overall, clarity proved less variable than other dimensions, as most tools delivered interpretable output once results were generated, but differences in labelling, visual structure, and presentation determined how quickly and reliably analysts could extract insights.

Scoring differences are further illustrated by Q3 (variant with the longest average execution time), both Apromore and Disco presented tabular results sorted by duration. However, Apromore reported average durations in decimal minutes, requiring the analyst to mentally convert values to seconds, whereas Disco displayed durations directly in a standard time format (minutes and seconds). This difference is explicitly noted in the evaluation records and explains why Apromore received a clarity score of 4, while Disco received a score of 5 for the same task.

Apromore generally required the fewest clicks, as it automatically restricted logs to complete transitions. Disco generally fell in the middle, requiring manual filtering, while ProM demanded the most interactions, sometimes more than twice as many as the other tools. For example, in Q7 (cases longer than twice the average duration), Apromore required six clicks, Disco fifteen, and ProM twenty-six. These results show that effort can diverge substantially even when the clarity scores are similar. Tasks that required more user interactions tended to score lower in ease of use. ProM required more than twice as many clicks in many tasks compared to Apromore, and this was consistently reflected in its lower usability ratings. Most tools produced interpretable output once generated, but differed in how smoothly they guided users to those results.

While minimal click counts provide a measurable proxy for interaction effort, they do not capture the full cognitive load imposed by a tool’s interface, as fewer interactions may still involve greater mental effort in less intuitive workflows. In the observed results, tools that required fewer interactions also tended to receive higher ease-of-use scores, suggesting more streamlined and guided workflows. In contrast, clarity scores showed relatively small variation across tools and did not display a consistent pattern with respect to interaction effort, indicating that result interpretability can remain high even when more interactions are required.

#### 5.2.2. Coverage and Consistency of Analytical Outcomes

This subsection addresses RQ3, which examines the consistency of the analytical results across the investigated tools using the same dataset. Although most of the outputs were aligned, differences appeared in variant counts and coverage of analytical questions. For example, Apromore reported 140 process variants, whereas ProM and Disco each reported 143. This discrepancy likely arises from differences in filtering rules. Apromore may exclude incomplete or very short traces, while ProM and Disco appear to count all distinct traces. These findings suggest that tool design choices influence the reported results and should be taken into account when comparing analyses across platforms. Beyond the differences discussed above, no further substantial discrepancies were observed in the analytical outcomes produced by the tools. Remaining variations were limited to minor differences, such as rounding of performance metrics (e.g., reported average case durations or activity execution times). For transparency, the complete set of analytical outputs obtained for each task and tool is provided in the online repository [28].

The evaluation also revealed differences in the analytical questions each tool was able to answer. Apromore returned results for nearly all tasks, missing Q5 (execution deviation) and Q11 (planned versus actual durations). Disco and ProM failed to answer four questions each. Although two of these overlapped, the remaining gaps were different, indicating that their limitations are partly overlapping but not identical. Q11 proved to be unsolvable using the built-in functionalities of all three tools. Although the event log contained the necessary columns for planned-versus-actual comparisons, the required data transformations were not available within the tools and would need to be performed as external preprocessing before import. This case is therefore best characterised as unsupported by built-in functionality. We note that, although such preprocessing could potentially enable the analysis, this was not implemented or empirically validated in the study. Q5 was only answered by ProM, despite its lower usability, illustrating that algorithmic depth can at times compensate for interface limitations. In Q9 (conformance checking), Apromore was able to produce compliance percentages, but only after adapting the BPMN model to account for repeated activity names. In contrast, Disco and ProM offered no direct support for conformance checking against the provided BPMN model. These results suggest that the analytical coverage and reported results are not entirely consistent across tools. No single tool was capable of handling the full spectrum of typical analytical questions defined in the study.

### 5.3. Comparison with Related Work

Several studies have examined process mining tools, and their results provide valuable context for interpreting our findings. Kesici et al. [2] emphasise that additional features beyond the core process mining functionalities depend on enterprise needs. Their observation aligns with our finding that the investigated tools do not offer direct integration with IoT data, highlighting how the relevance of specific capabilities is highly dependent on the context and the practitioner’s perspective.

A recurring theme in related work is the contrast between Disco and ProM in terms of usability. Kesici et al. [2] note that ProM is “not easy for beginners”, while Disco is considered easier to use. A study in optimizing healthcare processes [31] similarly identifies ease of use as a key criterion. Gomes et al. [32] further highlight Disco as the simplest and most intuitive tool, while acknowledging that both Disco and ProM allow complete analysis without deep theoretical knowledge, although with limitations in customization. These perspectives broadly support our evaluation, which ranked Disco higher than ProM in usability. However, our results diverge from those of Gomes et al. [32] in that we found ProM significantly less intuitive for analytical IoT-oriented tasks.

Another consistent finding across the literature concerns conformance checking and advanced analytical features. Gomes et al. [32] report that Disco performs poorly in enhancement and conformance checking, whereas ProM proved effective in this respect. This strongly supports our finding that Disco lacked conformance checking support, but it diverges in the case of ProM. In our task-based evaluation, ProM was less effective when applied to checking conformance to the provided process model, which highlights how evaluation outcomes can depend on the chosen dataset. Parente et al. [33] also note that ProM supports conformance checking but caution that its extensive plug-in ecosystem and limited documentation create accessibility challenges. This resonates with our observation that ProM’s analytical breadth comes at the expense of usability.

Although most comparative studies provide detailed commentary on Disco and ProM, Apromore is under-represented in narrative discussions. Loyola-González [16] includes Apromore in their feature taxonomy, where it scores competitively in a wide range of functional categories. In particular, Apromore shows stronger coverage of monitoring, dashboards, and advanced charting than Disco, and comparable support for process analysis tasks such as benchmarking and variant breakdown. These strengths are consistent with our task-based evaluation, where Apromore was the only tool to support KPI tracking, dashboards, and automated reporting, all of which are essential for IoT-related continuous monitoring.

### 5.4. Limitations

The following limitations should be acknowledged when interpreting the findings. The reported capabilities, as analysed from vendor materials, reflect what the vendors claim, rather than what is necessarily delivered in practice. Some advertised features may prove less flexible or effective during real-world use, while undocumented capabilities may exist but remain uncaptured in the review. Despite this, analysis provides a systematic and transparent way to map functional capabilities, supporting reproducibility between tools.

Beyond the analysis of vendor materials, task-based evaluation also introduces contextual limitations. The analysis was conducted using a single dataset from a smart factory case and was evaluated against three tools, as explained in Section 3. This restricts the generalisation of the results, as other datasets or process mining tools might yield different results. The study did not include scalability or performance tests, as this would require a dedicated experimental setup and larger datasets. These aspects remain important directions for future research, although meaningful scalability evaluations will only be possible once tools offer more comprehensive support for IoT-specific data and processes.

The evaluation inevitably contains subjective elements, as qualitative scoring requires expert interpretation. To limit this influence, we used structured templates, predefined scoring criteria, and required written justifications for every score. Assessments were subsequently audited by a senior expert, providing an additional layer of validation and ensuring consistency between tools and functional capabilities. Future studies should apply the framework to additional IoT datasets as these become available and to a wider set of process mining tools, while also involving multiple expert evaluators to assess inter-rater reliability and strengthen external validity. To illustrate the applicability of the proposed methodology across different IoT application scenarios, the same evaluation procedure could be applied to process-level event logs derived from other IoT-enabled systems, such as smart cities or sports monitoring environments. In line with the scope of this study, such datasets would assume that sensor-to-event abstraction and data integration are handled upstream, and that the process mining tools operate on structured event logs. While the functional capability assessment would remain unchanged, the analytical questions of the task-based evaluation would need to be adapted to the structure and semantics of sensor-derived event logs reflecting physical system behaviour.

Another limitation concerns the pace of technological development. Process mining tools evolve rapidly, meaning that evaluations can become outdated shortly after they are conducted. The findings presented here should be interpreted as a time-specific snapshot of the functional capabilities described at the time of the review rather than as a permanent assessment. Large language model (LLM)-based functionalities could not be included in the evaluation, as they were not available under the academic licenses used.

## 6. Conclusions

This study compared three well-established process mining tools through a structured framework that combined analysis of vendor materials with a controlled task-based evaluation on smart factory event logs. The results showed that while all tools support core process discovery, their effectiveness diverges in conformance checking, performance analysis, and usability. Apromore demonstrated broad functional coverage and the highest ease of use, Disco provided strong discovery features but limited conformance support, and ProM offered algorithmic depth at the expense of usability and interaction effort. In particular, none of the tools in our evaluation was able to answer all 13 analytical questions defined in the task-based evaluation. Table 10 provides a condensed overview of the comparative findings for the evaluated process mining tools, from the perspective of an IoT engineer. It summarises the main advantages and disadvantages, serving as a high-level synthesis of the findings presented in Section 4. The table is not intended to replace the detailed analysis discussed in Section 5, but rather to highlight key differences in usability, functionality, and IoT relevance.

For IoT practitioners, these findings suggest that tool selection should be guided by task scope and usability requirements. Based on our evaluation, Apromore appears to be best suited for analysis where conformance and monitoring are essential, while Disco proved effective for lightweight exploration and the rapid discovery of process models. ProM remains valuable in research and experimental contexts, but appears less appropriate for engineers who require intuitive interfaces and efficient workflows. Importantly, the evaluated tools continue to rely on preprocessed event logs, meaning that IoT engineers must still manually bridge the gap between raw sensor data and process-level abstractions. Accordingly, the findings should be interpreted as an assessment of process mining tool support after IoT data has been transformed into event logs, rather than as an evaluation of the complete IoT data pipeline from raw sensor streams to process insights.

The evaluated tools showed limited adaptation to sensor-rich and real-time contexts. Although other tools may address these needs, our results demonstrate that even leading tools, such as Apromore, listed in Gartner’s 2025 Magic Quadrant [12], still require substantial preprocessing and lack direct IoT integration. This indicates that further benchmarking is necessary across a broader range of tools and datasets before generalisations can be made. The evaluation methodology developed in this study is applicable to other IoT-enabled domains beyond smart manufacturing. While the functional capability assessment remains domain-agnostic, the task-based questions would require adaptation to reflect the domain-specific analytical priorities and the semantic structure of sensor-derived event logs (see Section 5.4 for an illustrative example).

Several directions for future research emerge from this study. First, scalability should be tested with larger and more complex IoT event logs to determine whether performance degrades under the data volumes typical of industrial settings. Second, evaluations should be expanded to include additional datasets as they become more readily available in the future. Third, future work should investigate the adoption of emerging standards such as OCEL 2.0, which enables object-centric process mining. Unlike traditional case-based logs, OCEL 2.0 can represent many-to-many relationships between machines, sensors, and products that characterise IoT environments, making it a promising direction for integrating process mining with IoT [34,35]. Pursuing these research directions depends on the availability of suitable IoT datasets and on process mining tools adopting these emerging standards, as current limitations in both data and tooling constrain the extent to which such investigations can be realised. In addition, future studies could examine the role of LLM-based functionalities in process mining tools, focusing on how they support analytical tasks such as query formulation, interpretation of results, and user guidance.

Our results also suggest a relationship between interaction effort and ease of use, with tools requiring more clicks generally scoring lower in usability. This interaction could be further validated in future studies through multi-user evaluations and formal usability testing frameworks. Such studies would also enable the inclusion of task execution time as a complementary effort metric. While time-based measures offer additional insight into task complexity, they are sensitive to factors such as prior tool familiarity and learning effects, which require carefully controlled experimental conditions to isolate, as described in more detail in Section 3.3.2. The task-based evaluation approach complements quantitative interaction metrics with qualitative assessments that capture workload imposed by the tool and the clarity of its outputs. Process mining is more likely to become a practical tool for IoT engineers if sophisticated analyses can be performed with minimal effort and without specialised expertise [11].

## Figures and Tables

**Table 1:** Six functional areas derived from the literature represent the core capabilities of process mining tools.

Functional Area	Description
Data Management	Covers data ingestion, transformation, data cleaning, and preparation for analysis.
Process Discovery	Translates raw event logs into visual and interpretable process models.
Conformance Checking	Compares observed processes with reference models to detect deviations.
Process Analysis	Supports bottleneck detection, performance analysis, and inefficiency identification.
Monitoring and Reporting	Enables continuous tracking of process metrics and customizable reporting.
Support	Includes documentation, user guides, training, and community or commercial support.

**Table 2:** Ease of use scoring criteria.

Score	Description
1	Very hard to use. Tasks require a high level of cognitive effort and technical knowledge. The interface is obscure, poorly labelled, or inconsistent, forcing trial and error.
2	Difficult. Tasks require considerable effort due to confusing navigation, unclear labels, or inconsistent workflows. Users struggle to locate or apply functions.
3	Moderate. Tasks are achievable but involve noticeable cognitive effort. Some elements are intuitive, but others are inconsistently labelled or non-obvious, creating a learning curve.
4	Easy to use. Tasks can be completed with little cognitive effort. The interface is well-labelled and mostly consistent, with clear workflows for common functions.
5	Very easy. Tasks can be completed almost effortlessly. The interface is intuitive, consistent, and self-explanatory, minimising friction at every step.

**Table 3:** Criteria for clarity scoring.

Score	Description
1	Output is hard to read or interpret. Poor or missing labels. Structure is confusing. An analyst needs outside help or trial and error to make sense of it.
2	Output is somewhat readable but lacks detail or context. Labels or visuals are inconsistent or vague. An analyst can understand it with effort, but may misinterpret parts.
3	Output is generally understandable. Basic labels and structure are in place. Some effort is still needed to extract key insights, especially in complex cases.
4	Output is logically structured, well-labelled and easy to follow. An analyst can interpret key insights without extra help. Some minor improvement in explanation or layout could help.
5	Output is immediately understandable. Clear labels, helpful tooltips or legends, and strong structure. No extra effort needed. Supports direct decision-making.

**Table 4:** Criteria for functional capabilities.

Score	Description
0	No support. The tool does not offer this functionality in any form.
1	Minimal functionality. Limited scope with almost no flexibility. Suitable only for a single, narrow use case.
2	Limited functionality with some flexibility. Can cover standard needs but lacks depth, consistency, or adaptability.
3	Moderate functionality with reasonable coverage and basic configurability. Provides solid support for typical use cases but struggles with more complex or specialised scenarios.
4	Strong functionality with flexibility and user-configurable options. Provides reliable support for the most common and some complex use cases.
5	Advanced functionality with broad coverage and adaptability. Supports complex and varied scenarios, including those beyond typical use cases.

**Table 5:** Analytical questions are used in the task-based evaluation.

ID	Analytical Question
Q1	What is the most frequent non-failing path that includes at least two activities (excluding start/end)?
Q2	How many different process variants exist in the smart factory?
Q3	Which variant has the longest average execution time (with at least two cases)?
Q4	What is the average number of activities per case?
Q5	Which activity shows the highest deviation in execution duration across cases?
Q6	What is the average completion time of a case?
Q7	How many cases last longer than twice the average case duration?
Q8	Which is the longest path for a case?
Q9	To what extent do cases under the process model WF_101 comply with WF_101?
Q10	What are the most common activities and how often do they occur?
Q11	How often do actual durations exceed planned durations across activities?
Q12	Which activity has the highest total execution time?
Q13	Which resources cause the most failures?

**Table 6:** Functional capabilities scores based on analysis of vendor materials.

	Apromore	Disco	ProM
Data Management	5	4	3
Process Discovery	5	5	5
Conformance Checking	5	1	4
Process Analysis	5	4	4
Monitoring and Reporting	5	2	1
Support	5	5	2

**Table 7:** Clarity scores across tools.

Question	Apromore	Disco	ProM
Q1	5	5	5
Q2	5	4	4
Q3	4	5	/
Q4	5	/	5
Q5	/	/	5
Q6	5	5	4
Q7	5	4	4
Q8	5	5	3
Q9	5	/	/
Q10	5	5	4
Q11	/	/	/
Q12	5	5	/
Q13	5	5	3

**Table 8:** Number of clicks done in the tool to obtain the answer.

Question	Apromore	Disco	ProM
Q1	10	21	25
Q2	2	3	15
Q3	4	12	/
Q4	6	/	13
Q5	/	/	25
Q6	2	12	17
Q7	6	15	26
Q8	4	11	18
Q9	8	/	/
Q10	4	11	14
Q11	/	/	/
Q12	5	11	/
Q13	10	18	17

**Table 9:** Ease of use scores.

Question	Apromore	Disco	ProM
Q1	5	4	2
Q2	5	4	3
Q3	5	4	/
Q4	5	/	3
Q5	/	/	2
Q6	5	5	3
Q7	5	4	2
Q8	5	5	3
Q9	4	/	/
Q10	5	5	4
Q11	/	/	/
Q12	5	5	/
Q13	5	5	2

**Table 10:** Summarised findings of the evaluated tools on the Smart Factory dataset from an IoT engineer’s perspective.

Dimension	Apromore	Disco	ProM
Ease of Use	Very high (intuitive GUI, guided workflows)	High (simple interface, embedded help)	Low (complex menus, high learning curve)
Effort (interactions)	Fewest clicks	Moderate	Most clicks
Clarity of output	Excellent (clear labels, structured visuals)	High	Moderate (less intuitive in some cases)
Functional breadth	Broad (discovery, dashboards, conformance, KPIs)	Moderate (basic conformance and monitoring)	Very broad (many plug-ins, but fragmented, absent monitoring)
Support	Excellent	Excellent	Limited
Conformance checking with provided BPMN	Supported (after model adjustment)	Not supported	Not supported
Real-time/IoT integration	Moderate (available S3 connector, data preprocessing needed)	None	None
Reporting and monitoring	Automated dashboards, KPIs	Static exports	Static exports, plug-in dependent
Main strengths	Usability, breadth, monitoring	Simplicity, quick discovery	Depth, extensibility
Main limitations	Delayed interactions caused by cloud processing	Limited conformance	Poor usability, fragmented UX

## Data Availability

The dataset supporting the findings of this study is publicly available in the Zenodo repository (DOI: https://doi.org/10.5281/zenodo.17119904).

## Abbreviations

The following abbreviations are used in this manuscript:

IoT	Internet of Things
BPMN	Business Process Model Notation
ID	Identifier
MES	Manufacturing Execution System
ERP	Enterprise Resource Planning
XES	eXtensible Event Stream
AI	Artificial Intelligence
KPI	Key Performance Indicator
LLM	Large Language Model
UI	User Interface
